# Endotracheal Tube Securement and Medical Device-Related Pressure Injury Incidence in the Intensive Care Unit: A Single-Center Retrospective Cohort Study

**DOI:** 10.7759/cureus.100303

**Published:** 2025-12-29

**Authors:** Yutaro Momoeda, Kentaro Hara, Yukiko Nakamura, Yoshihumi Kubota, Chison Gon, Chikaaki Nakamichi, Masaki Fujioka

**Affiliations:** 1 Department of Nursing, National Hospital Organization Nagasaki Medical Center, Nagasaki, JPN; 2 Department of Fundamental Nursing, Faculty of Life Sciences, Kumamoto University, Kumamoto, JPN; 3 Clinical Research Center, National Hospital Organization Nagasaki Medical Center, Nagasaki, JPN; 4 Healthcare Management Research Center, Chiba University, Chiba, JPN; 5 Department of Emergency and Critical Care Medicine, National Hospital Organization Nagasaki Medical Center, Nagasaki, JPN; 6 Department of Plastic and Reconstructive Surgery, National Hospital Organization Nagasaki Medical Center, Nagasaki, JPN

**Keywords:** endotracheal tube, endotracheal tube fastener, intensive care unit, medical device-related pressure injuries, pressure injury prevention

## Abstract

Background

Medical device-related pressure injuries (MDRPIs) are common among mechanically ventilated patients in the intensive care unit (ICU) and are frequently associated with endotracheal tubes. This study aimed to evaluate the association between different endotracheal tube securement methods and the occurrence of MDRPIs among mechanically ventilated patients.

Methodology

This single-center, retrospective cohort study was conducted at an advanced critical care and emergency center in Japan. Patients aged ≥16 years who required invasive mechanical ventilation for ≥48 hours between April 2020 and November 2024 were included. The AnchorFast SlimFit (Hollister) device was introduced in 2022 as an alternative to adhesive tape and routinely used since 2023. The incidence of MDRPIs (including intraoral sites) was compared between securement methods.

Results

A total of 364 patients were analyzed (AnchorFast group, n = 163; adhesive tape group, n = 201). The overall incidence of MDRPIs was significantly lower in the AnchorFast group than in the adhesive tape group (25/163 (15.3%) vs. 65/201 (32.3%), p < 0.001). Lip-related MDRPIs were less frequent in the AnchorFast group (8/163 (4.9%) vs. 39/201 (19.4%), p < 0.001). Multivariate logistic regression analysis identified AnchorFast use as an independent factor associated with a lower risk of MDRPIs occurrence (odds ratio = 0.522; 95% confidence interval = 0.033-0.626; p = 0.031).

Conclusions

AnchorFast fixation was associated with a markedly lower incidence of MDRPIs than conventional tape, particularly on the lips. Given the observational design, causal inference is limited, and prospective studies are warranted.

## Introduction

Medical device-related pressure injuries (MDRPIs) are defined as localized injuries to the skin and/or underlying tissue resulting from pressure and/or shear exerted by medical devices [1,2]. MDRPIs are iatrogenic ulcers caused not by body weight but by sustained device-related pressure, making their prevention a critical area of focus [3,4]. The Japanese Society of Pressure Ulcers has identified MDRPI prevention as a key action plan, and in recent years, efforts to prevent and manage MDRPIs have gained attention in Japan [5].

Critically ill patients admitted to intensive care units (ICUs) are at particularly high risk for MDRPIs due to a combination of factors, including physiological instability and the use of multiple medical devices [6,7]. Previous studies have reported that 11.6% to 30.6% of ICU patients develop MDRPIs, with the most common cause being endotracheal tubes, accounting for 37.5% to 61.4% of cases [8-10]. Therefore, implementing preventive strategies for MDRPIs in mechanically ventilated ICU patients is of paramount importance.

At our advanced critical care and emergency center, the conventional tape-based endotracheal tube securement using adhesive elastic bandages (Silkytex®, ALCARE Co., Ltd., Tokyo, Japan) was replaced in 2022 by a trial introduction of the AnchorFast™ endotracheal tube securement device (Hollister Incorporated, Libertyville, IL, USA). Since 2023, patients expected to require mechanical ventilation for more than 48 hours have been managed using the AnchorFast for endotracheal tube securement. The AnchorFast system adheres to both cheeks via hydrocolloid pads, secures the tube with an integrated clamp, and allows lateral adjustment along a track rail. A randomized controlled trial has reported that AnchorFast reduces the incidence of MDRPIs on the lips and face compared with traditional tape methods [11].

However, MDRPIs can also occur in the oral mucosa, such as the tongue and palate, due to pressure from the endotracheal tube or contact with bite blocks [12,13]. Recent studies report that the incidence of oral mucosal MDRPIs ranges from 0.8% to 30.4% [14]. To date, few studies have investigated the impact of different securement methods, particularly tape-based methods, on the occurrence of oral mucosal MDRPIs [15,16], and an effective securement approach to prevent endotracheal tube-related MDRPIs has not yet been clearly established.

Moreover, most of these findings originate from international studies. In Japan, existing research has primarily focused on the safety of oral care and nursing workload efficiency when using endotracheal tube fasteners [17,18]. Therefore, this study aimed to evaluate the association between the use of endotracheal tube fasteners and the occurrence of MDRPIs, with the incidence of MDRPIs as the primary outcome, among mechanically ventilated patients admitted to our ICU.

## Materials and methods

Study design

This single-center, retrospective cohort study was conducted at an advanced critical care and emergency center in Japan. The study evaluated the association between different endotracheal tube securement methods and the occurrence of MDRPIs among mechanically ventilated ICU patients. Data were retrospectively extracted from electronic medical records covering April 1, 2020, to November 30, 2024 (analysis periods defined below). The study was designed and reported in accordance with the Strengthening the Reporting of Observational Studies in Epidemiology (STROBE) guidelines [19]. Because of the retrospective nature of the study, no a priori sample size calculation was performed. Instead, all consecutive patients who met the eligibility criteria during the study period were included in the analysis to minimize selection bias.

Study period and participants

The study population included patients aged ≥16 years who were admitted to the Advanced Critical Care and Emergency Center and underwent invasive mechanical ventilation for ≥48 hours between April 1, 2020, and November 30, 2024. Exclusion criteria were patients aged ≤15 years; patients who underwent mechanical ventilation for <48 hours; patients transferred from other hospitals who were already intubated upon admission; patients with facial trauma or facial burns; patients who received mechanical ventilation via nasotracheal intubation; and patients admitted during the pilot introduction period of the AnchorFast device (April 1, 2022, to March 1, 2023).

Exposure definition and groups

Endotracheal tube securement practice changed unit-wide during the study window. Patients admitted during April 1, 2020, to March 31, 2022 (pre-implementation) received adhesive-tape securement (Tape group), whereas patients admitted during April 1, 2023, to November 30, 2024 (post-implementation) were managed with the AnchorFast Slim™ securement device (AnchorFast group). Admissions from April 1, 2022, to March 31, 2023, were excluded to avoid contamination from the pilot introduction. Group membership reflected the calendar period and routine practice; no investigator allocation occurred.

Exclusion of the trial introduction period

The one-year period from April 2022 to March 2023, during which AnchorFast was trial-introduced, was excluded from the analysis to minimize observer and performance bias associated with variability in nurses’ securement techniques. A national survey of ICU nurses in Japan regarding oral care for intubated patients had identified endotracheal tube securement as the technique in which nurses felt the least confident [20]. To ensure standardization of the AnchorFast securement technique, this transitional period was established. During the trial period, nurses received product handling instructions from the manufacturer, developed usage manuals, and underwent on-the-job training with nurses who had received specialized instruction before formal implementation.

Tape group (pre-implementation)

In the Adhesive Tape group, the endotracheal tube was secured to the upper jaw using adhesive elastic bandages (Silkytex®, ALCARE Co., Ltd., Tokyo, Japan). The securement tapes were replaced once daily, and the tube position was alternated between the left and right sides. For skin protection, a thin hydrocolloid dressing (DuoACTIVE® ET, ConvaTec Inc., Bridgewater, NJ, USA) was applied to the cheeks under the tape. When a bite block was used, a B-BOC® device (NIPRO Corporation, Osaka, Japan) designed for external placement of the endotracheal tube was employed.

AnchorFast group (post-implementation)

In the AnchorFast group, the endotracheal tube was secured using the AnchorFast Slim™ device (AnchorFast) (Hollister Incorporated, Libertyville, IL, USA). When a bite block was required, the same B-BOC® device (NIPRO Corporation, Osaka, Japan) designed for external tube placement was used. To relieve pressure, the tube position was adjusted every two hours. If the securement remained secure, the device was used continuously for up to seven days.

Data collection and variables

Patient data were retrospectively collected from electronic medical records. The primary outcome was the occurrence of MDRPIs attributable to the endotracheal tube/holder. Secondary outcomes included the anatomical distribution of MDRPI sites and days from intubation to MDRPI onset. Baseline and clinical variables included age, sex, body mass index (BMI), primary diagnosis, route of ICU admission (emergency department vs. general ward), duration of oral intubation, Acute Physiology and Chronic Health Evaluation II (APACHE II) score, vasopressor use (norepinephrine), diabetes mellitus, bite-block use, ICU length of stay, and laboratory values at ICU admission (albumin, total protein, C-reactive protein).

Assessment of MDRPIs

The presence or absence of MDRPIs was initially identified based on the documentation of skin and mucosal findings in daily nursing records completed by the primary nurse. Initial screening assessments were performed by the responsible nurse during routine care. When an MDRPI was suspected, a secondary evaluation was conducted by a Certified Nurse in Wound, Ostomy, and Continence Nursing [21], who performed a detailed clinical assessment and documented the findings. All suspected MDRPIs were subsequently reported to and reviewed by a plastic surgeon, who made the final diagnosis. The depth and stage of MDRPIs were classified according to the staging system established by the National Pressure Injury Advisory Panel [22].

Statistical analysis

Categorical variables were compared using the chi-square or Fisher’s exact test, as appropriate. For continuous variables, distributional assumptions were assessed; t tests were used for approximately normal data, and Mann-Whitney U tests otherwise. Statistical significance was set at p-values <0.05 (two-sided). To analyze the association between securement method and MDRPIs occurrence, multivariable logistic regression was performed with MDRPIs occurrence (yes/no) as the dependent variable. Clinically relevant confounders (age, sex, BMI, APACHE II score, vasopressor use, diabetes, duration of oral intubation, bite-block use) were included a priori [23]. ICU length of stay was summarized descriptively and used in sensitivity analyses to avoid over-adjustment. Outliers were defined as values below Q1-1.5×IQR or above Q3+1.5×IQR. All analyses were conducted using JMP® version 18 (SAS Institute Inc., Cary, NC, USA).

Ethical considerations

This study was approved by the Institutional Ethics Committee of our hospital (approval number: 2024040). As this was a retrospective observational study using anonymized data, the requirement for individual informed consent was waived in accordance with institutional policy. The study was conducted in accordance with the ethical principles of the Declaration of Helsinki and relevant guidelines for epidemiological research.

## Results

Participant characteristics

**Figure 1:**
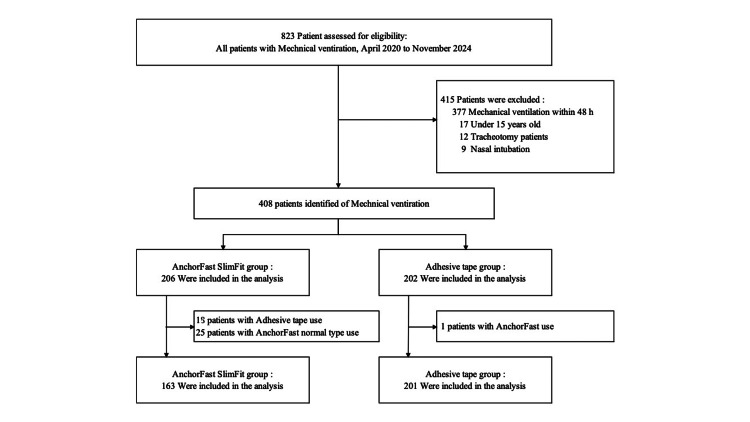
Study flowchart. A total of 823 mechanically ventilated patients were screened, and 415 were excluded based on predefined criteria. Among the remaining 408 patients, exclusions due to mismatched securement devices resulted in 163 patients in the AnchorFast SlimFit group and 201 in the adhesive tape group for the final analysis.

During the study period, 823 patients were admitted to the Advanced Critical Care and Emergency Center and received mechanical ventilation. Of these, 415 patients met the exclusion criteria. In addition, 43 patients in the AnchorFast group and one patient in the Adhesive Tape group were excluded due to differences in securement methods. The final analysis included 364 patients: 163 in the AnchorFast group and 201 in the Adhesive Tape group (Figure 1).

Patient characteristics are presented in Table 1. The cohort included 224 male and 140 female patients, with mean ages of 73 years in the AnchorFast group and 71 years in the Adhesive Tape group. The most common primary diagnosis in both groups was neurological disease (71/163 (43.6%) vs. 92/201 (45.8%), p = 0.021). Respiratory failure was significantly more prevalent in the AnchorFast group (33/163 (20.3%) vs. 21/201 (10.5%), p = 0.021). The mean ICU length of stay was 12 days in both groups, and the mean duration of intubation was seven days in the AnchorFast group and nine days in the Adhesive Tape group. A high proportion of patients in both groups were managed with bite blocks (140/163 (85.9%) vs. 169/201 (84.1%), p = 0.630).

**Table 1:** Baseline characteristics of patients in the AnchorFast SlimFit and Adhesive Tape groups. Baseline characteristics of patients in the AnchorFast SlimFit and adhesive tape groups are presented as n (%) for categorical variables and median (IQR) for continuous variables. P-values were calculated using chi-square or Mann–Whitney U tests, as appropriate. *: Significant at p < 0.05; **: p < 0.01. IQR = interquartile range; PCAS = post–cardiac arrest syndrome; APACHE II = Acute Physiology and Chronic Health Evaluation II; CRP = C-reactive protein

Variables	Group		P-value
	AnchorFast SlimFit (n = 163)	Adhesive tape (n = 201)	
Gender			0.473
Male	97 (59.5%)	127 (63.2%)	
Female	66 (40.5%)	74 (36.8%)	
Age (median, IQR)	73 (60−80)	71 (59−80.5)	0.831
Admission route			0.002**
ER	122 (74.9%)	175 (87.1%)	
Ward	41 (25.1%)	26 (12.9%)	
Diagnostic category			0.021^＊^
Neurologic	71 (43.6%)	92 (45.8%)	
Sepsis	12 (7.36%)	32 (15.9%)	
Trauma	7 (4.3%)	10 (5.0%)	
Respiratory failure	33 (20.3%)	21 (10.5%)	
Cardiovascular	8 (4.9%)	13 (6.5%)	
PCAS	11 (6.8%)	6 (3.0%)	
Others	21 (12.9%)	27 (13.4%)	
ICU length of stay, days (median, IQR)	12 (8−15)	12 (8−16)	0.168
Duration of intubation, days	7 (5−10)	9 (5−11.5)	0.111
Tracheostomy, n (%)			0.327
Yes	74 (45.4%)	81 (40.3%)	
No	89 (54.6%)	120 (59.7%)	
Vasopressors			0.685
Used	36 (22.1%)	48 (23.9%)	
Not used	127 (77.9%)	153 (76.1%)	
Bite-block			0.630
Use	140 (85.9%)	169 (84.1%)	
Not use	23 (14.1%)	32 (15.9%)	
APACHE II score	19 (14–23)	19 (16–25)	0.050
Diabetes	30 (18.5%)	38 (19.0%)	0.907
Body mass index	22.2 (20−25.5)	22.8 (20.3−25.6)	0.467
Total protein (g/dL)	6.7 (5.8−7.3)	6.7 (5.9−7.3)	0.967
Albumin (g/dL)	3.5 (2.8−4.1)	3.4 (2.8−4)	0.737
CRP (mg/dL)	0.31 (0.08−7.32)	0.75 (0.12−6.26)	0.927

Incidence of MDRPIs and associated factors

The incidence of MDRPIs by securement method is summarized in Table 2. MDRPIs occurred in 90 of the 364 patients overall, including 25 cases in the AnchorFast group and 65 cases in the Adhesive Tape group. The incidence of MDRPIs was significantly lower in the AnchorFast group compared with the Adhesive Tape group (25/163 (15.3%) vs. 65/201 (32.3%), p = 0.001).

**Table 2:** Comparison of MDRPI incidence, time to onset, and lesion location between the AnchorFast SlimFit and Adhesive Tape groups. **: Significant at p < 0.01. MDRPIs = medical device–related pressure injuries

Outcome	Group		P-value
	AnchorFast SlimFit (n = 163)	Adhesive tape (n = 201)	
Number of MDRPIs			0.001**
Yes	25 (15.3%)	65 (32.3%)	
No	138 (84.7%)	136 (67.7%)	
Intubation to MDRPIs, days	5 (2.5−6)	4 (2−6)	0.977
Location of MDRPIs			
Lip	8 (4.91)	39 (19.4%)	0.001**
Tongue	10 (6.2%)	19 (9.5%)	0.247
Palate	4 (2.5%)	11 (5.5%)	0.189
Gum	1 (0.6%)	7 (3.5%)	0.079
Cheek	2 (1.2%)	0 (0.0%)	0.198

When limited to extraoral sites, for example, the lips and facial skin, the incidence of MDRPIs was 6.13% in the AnchorFast group and 19.4% in the Adhesive Tape group. The most frequent MDRPIs site was the lips, with 47 cases (8 in the AnchorFast group vs. 39 in the Adhesive Tape group, p = 0.001), followed by the tongue (10 vs. 19), palate (4 vs. 11), gingiva (1 vs. 7), and cheeks (2 vs. 0). The incidence of lip-related MDRPIs was significantly lower in the AnchorFast group.

The median number of days from intubation to MDRPIs onset was five days (IQR = 2.5-6) in the AnchorFast group and four days (IQR = 2-6) in the Adhesive Tape group.

Logistic regression analysis

Results of the logistic regression analysis are shown in Table 3. The occurrence of MDRPIs was the dependent variable, and clinically relevant predictors, including AnchorFast use, age, sex, APACHE II score, duration of intubation, ICU length of stay, use of vasopressors, and serum albumin levels, were included as independent variables. Use of the AnchorFast device was independently associated with a reduced risk of MDRPIs (odds ratio = 0.522, 95% CI = 0.033-0.626, p = 0.031).

**Table 3:** Multivariable logistic regression analysis for factors associated with MDRPI occurrence. This table presents the results of multivariable logistic regression analysis to identify factors independently associated with the occurrence of MDRPIs. The use of AnchorFast SlimFit was significantly associated with a lower risk of MDRPIs compared to adhesive tape (β = 0.324, 95% CI = 0.033–0.626, p = 0.031). *: Statistically significant at p  < 0.05. MDRPIs = medical device–related pressure injuries; CI = confidence interval; APACHE II = Acute Physiology and Chronic Health Evaluation II; ICU = intensive care unit

Variable	Beta	Standard error	95%Cl	P-value
AnchorFast SlimFit (Adhesive tape)	0.324	0.150	0.033−0.626	0.031*
Age	-0.0009	0.010	-0.020−0.019	0.925
Sex (male)	-0.074	0.155	-0.378−0.233	0.633
APACHE Ⅱ	0.019	0.023	-0.026−0.064	0.412
Duration of mechanical ventilation	0.056	0.038	-0.019−0.133	0.142
ICU length of stay	0.045	0.031	-0.015−0.108	0.143
Vasopressors (not used)	-0.290	0.186	-0.655−0.080	0.120
Albumin	0.209	0.205	-0.185−0.623	0.307

## Discussion

Comparison with previous studies on MDRPI incidence

This study compared the incidence of MDRPIs associated with two endotracheal tube securement methods: conventional tape and the AnchorFast device. The results showed that the AnchorFast device significantly reduced the incidence of MDRPIs compared with the tape method. These findings are consistent with the randomized controlled trial by Landsperger et al., which reported lower rates of MDRPIs with an endotracheal tube fastener compared with adhesive tape [11].

Previous studies have reported the incidence of MDRPIs of 4-9% with the use of AnchorFast [22,23]. In our study, the overall incidence of MDRPIs in the AnchorFast group was 15.34%, which appears higher by comparison. However, earlier studies typically limited their investigation to perioral regions, for example, the lips, face, or ears, making direct comparison difficult. When restricted to extraoral MDRPIs (e.g., lips and face), our study found an incidence of 6.13% in the AnchorFast group, which is comparable to earlier reports. A unique feature of our study is that we also analyzed intraoral mucosal MDRPIs, which have been underrepresented in previous research, providing new insights into site-specific occurrences based on securement methods.

Site-specific effects of securement method on MDRPI prevention

This study also examined the distribution of MDRPI occurrence, including intraoral mucosal sites. AnchorFast securement was found to significantly reduce lip-related MDRPIs compared with the Adhesive Tape group. A notable feature of the AnchorFast system is the ability to adjust the tube position freely using a sliding shuttle clamp, allowing frequent shifts in pressure points. In contrast, the conventional tape method allowed position changes only once daily during tape replacement. The capacity for continuous repositioning likely contributed to the reduced incidence of MDRPIs on the lips, tongue, and palate in the AnchorFast group.

However, tongue-related MDRPIs were the most frequent in the AnchorFast group, accounting for 40% of MDRPI cases (10/25). This may indicate that in some cases, the adjustable function of the AnchorFast was not utilized effectively. According to Simone et al., challenges to repositioning endotracheal tubes include prone positioning, significant facial edema, patient agitation, and difficulty with oral access [24]. In our study population, many patients had neurological conditions requiring mechanical ventilation. Previous studies have reported a significant association between impaired consciousness and the development of oral mucosal injuries [25]. Many of these patients were in the postoperative period following craniotomy or had prolonged disturbances of consciousness, which may result in reduced spontaneous movement and sensory perception, as well as facial or tongue edema and restricted mouth opening. These conditions may limit appropriate repositioning of the endotracheal tube and thereby contribute to the development of tongue-related MDRPIs.

Our findings suggest that device-related factors alone are insufficient to prevent MDRPIs in all critically ill patients. Further care strategies specifically targeting intraoral mucosal health are needed. Previous research has shown that patients undergoing oral intubation have reduced moisture levels in the tongue and lips [26], and such dryness is associated with increased risk of device-related injury [27]. Therefore, implementing consistent oral moisturizing care to maintain a moist environment may be an essential preventive measure. Guidelines recommend four to six oral care interventions per day for intubated patients [28]; hence, determining optimal intervals for moisturizing care warrants further investigation.

Consideration of cost and practicality of securement methods

Although this study demonstrated the effectiveness of the AnchorFast device in reducing MDRPIs incidence, economic considerations are also critical for practical implementation. Adhesive Tape is relatively inexpensive and widely applicable across healthcare settings [16]. However, it requires daily tape changes, which may lead to increased cumulative costs. Additionally, tape changes typically require two nurses and may introduce variability in technique and workload burden [29].

In contrast, while AnchorFast entails higher initial costs, it can be used continuously for up to seven days if not loosened. This may reduce nursing workload, enhance safety, and standardize securement practices. Over time, the reduced need for frequent replacements could offset initial expenses. Moreover, as our study showed, the reduction in MDRPI incidence with AnchorFast could translate into lower resource use and decreased patient burden. Future studies should perform a comprehensive cost-effectiveness analysis comparing adhesive tape and AnchorFast, considering not only supply costs but also MDRPI prevention outcomes, nursing efficiency, and overall patient care impact.

Economic considerations for clinical implementation

From an economic perspective, although AnchorFast demonstrated a significant reduction in the incidence of MDRPIs in this study, its cost-effectiveness remains an essential consideration for clinical implementation. Conventional tape securement methods, for example, Silkytex®, are inexpensive (approximately $1.30 per roll), with an estimated seven-day cost of around $1.30 per patient assuming daily replacement. In contrast, the AnchorFast device (Hollister) has a higher initial cost (approximately $21.50 per unit), but it can be used continuously for up to seven days if properly secured, thereby reducing replacement frequency.

Beyond material costs, labor-related factors must also be considered. Tape securement often requires two nurses and involves time-consuming procedures, for example, alternating securement sides, applying skin barrier products, and cutting tape manually. These steps introduce variability and increase nursing workload. In comparison, AnchorFast offers a standardized design with a simple and reproducible application process, potentially enhancing procedural efficiency and reducing staffing demands.

Moreover, the occurrence of MDRPIs carries significant additional costs, including expenses for wound care materials, medications, and increased nursing time. Indirect costs, for example, patient discomfort, decreased quality of life, and prolonged ICU stays further compound the economic burden.

Considering these factors, although AnchorFast appears more costly initially, its potential to prevent MDRPIs, improve workflow efficiency, and reduce both direct and indirect healthcare costs suggests that it may represent a cost-effective preventive strategy in ICU settings. These findings highlight the value of evaluating not only material costs but also labor and outcome-related factors when selecting securement devices.

Limitations

This study has several limitations inherent to its retrospective observational design. First, the single-center, pre-post (calendar-period) comparison is susceptible to temporal confounding (e.g., changes in ICU case-mix, staffing, sedation, and oral care practices, or general pressure-injury prevention bundles) that may coincide with securement practice. Second, selection bias may have occurred during cohort assembly; it is possible that the clinical characteristics and trajectories of excluded or ineligible cases were associated with the development of MDRPIs, which may have influenced the study outcomes. Therefore, caution is warranted when attempting to generalize these findings beyond the studied population. Third, residual and unmeasured confounding cannot be excluded. Although we adjusted for clinically relevant variables, we did not capture some potential confounders, such as prone positioning, sedation depth and agitation, frequency/quality of oral care, facial or tongue edema severity, and device size/fit, which may influence MDRPI risk. Differences in time at risk may also persist despite adjustment for duration of intubation, because competing events (e.g., early extubation or death) can alter exposure time.

In addition, data collection relied exclusively on information extracted from medical records, raising the potential for information bias due to documentation inaccuracies or omissions. Outcome ascertainment depended initially on routine nursing documentation and was only subsequently verified by a wound, ostomy, and continence nurse when injury was suspected; thus, detection bias and site misclassification remain possible, particularly for intraoral lesions that are more difficult to visualize. These biases may have affected the accuracy of MDRPI detection and anatomical site classification, thereby limiting internal validity.

Finally, as this was a retrospective study, the sample size was determined by the number of eligible patients during the study period rather than by an a priori calculation. Missing data (if present) were handled by complete-case analysis, which may bias estimates if data were not missing at random. Cost data were not collected, precluding a formal economic evaluation.

To strengthen the evidence base, future studies should employ more robust designs, such as multicenter prospective cohorts and cluster randomized or stepped-wedge trials with standardized securement protocols, blinded outcome adjudication (including systematic intraoral assessment), and predefined process measures (e.g., repositioning frequency, oral care intervals). Full cost-effectiveness analyses comparing tape and device-based securement, incorporating supply costs, nursing time, and MDRPI-related resource utilization, are also warranted.

## Conclusions

This study demonstrated that the use of the AnchorFast endotracheal tube fastener significantly reduced the incidence of MDRPIs, particularly on the lips, compared with conventional tape securement in mechanically ventilated ICU patients. This benefit is probably due to the device’s ability to allow positional adjustments. However, intraoral MDRPIs, for example, tongue injuries, were still observed, especially in patients with neurological disorders. These findings suggest that securement devices alone may not be sufficient, and comprehensive oral care strategies are essential. Future studies should assess the cost-effectiveness of securement methods and investigate targeted oral care protocols for high-risk populations to further enhance MDRPI prevention in critical care settings.
